# Interstitial pneumonitis in a castration-resistant prostate cancer patient receiving cabazitaxel after thoracic radiation therapy: a case report

**DOI:** 10.1186/s12885-019-5942-4

**Published:** 2019-07-22

**Authors:** Yoshinori Yanai, Takeo Kosaka, Hiroshi Hongo, Mototsugu Oya

**Affiliations:** Department of Urology, Keio University School of Medicine, 35 Shinanomachi, Shinjuku-ku, Tokyo, 160-0016 Japan

**Keywords:** Cabazitaxel, Castration-resistant prostate cancer, Interstitial pneumonitis, Radiation recall pneumonitis, Thoracic radiotherapy

## Abstract

**Background:**

Interstitial pneumonitis is a rare reaction in a previously irradiated area of pulmonary or thoracic lesion after treatment with anticancer drugs such as taxanes.

**Case presentation:**

A 66-year-old man presented with a fever and dyspnea after treatment with cabazitaxel for castration-resistant prostate cancer. He was treated with an intravenous broad-spectrum antimicrobial agent, however he complained of dyspnea and had a pulse oximetric saturation of 80% while breathing room air. The patients had been treated for bone metastases with 37.5 Gy to the thoracic spine (Th 7) as a local radiotherapy. Radiological images showed pulmonary interstitial opacities in the irradiated field of the both lungs. The steroid pulse therapy was started. The patient’s dyspnea disappeared and the interstitial opacities had also improved.

**Conclusions:**

This report is a case of interstitial pneumonitis in a castration-resistant prostate cancer patient receiving cabazitaxel after thoracic radiation therapy.

## Background

Cabazitaxel is widely used for patients with castration-resistant prostate cancer. Local thoracic radiotherapy is also a common palliative radiation treatment. This report presents a case of interstitial pneumonitis after treatment with triggering agents such as taxanes, which is a rare reaction in a previously irradiated area of pulmonary or thoracic lesion [1].

## Case presentation

**Fig. 1:**
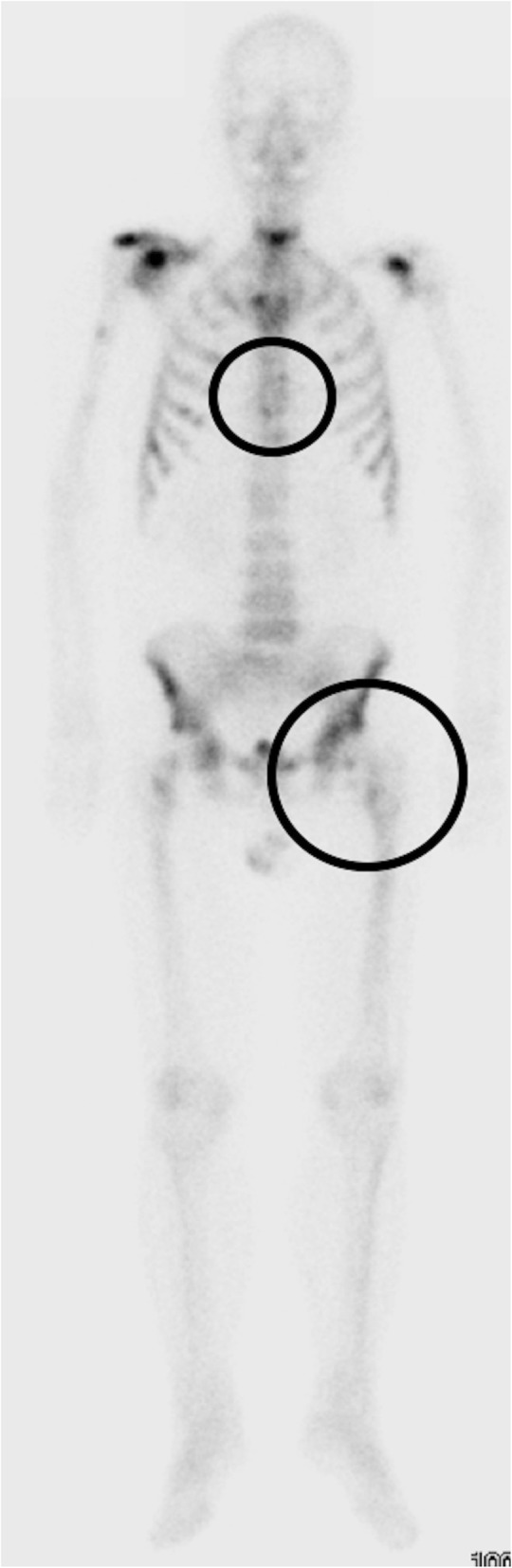
Bone scintigraphy shows multiple bone metastases, especially in the thoracic spine (Th7) and the left femur, where local therapy was performed

A 66-year-old man was diagnosed with castration-resistant prostate cancer (CRPC) and treated with enzalutamide followed by six cycles of docetaxel chemotherapy. However, his serum prostate specific antigen level increased to 899 ng/mL, so he was diagnosed as docetaxel resistant CRPC. Figure 1 shows bone metastases. The patient was treated for bone metastases with 37.5 Gy/15 fr to the thoracic spine (Th 7) as a local radiotherapy from February 2016 to March 2016. The radiation induced only mild dermatitis as an adverse event. Moreover, internal fixation of the left femur was performed to prevent pathological fractures due to metastasis to the left femur on August 2016. He also received six cycles of intravenous injections of radium-223 (50 kBq/kg) once every 4 weeks from September 2016 to January 2017.

**Fig. 2:**
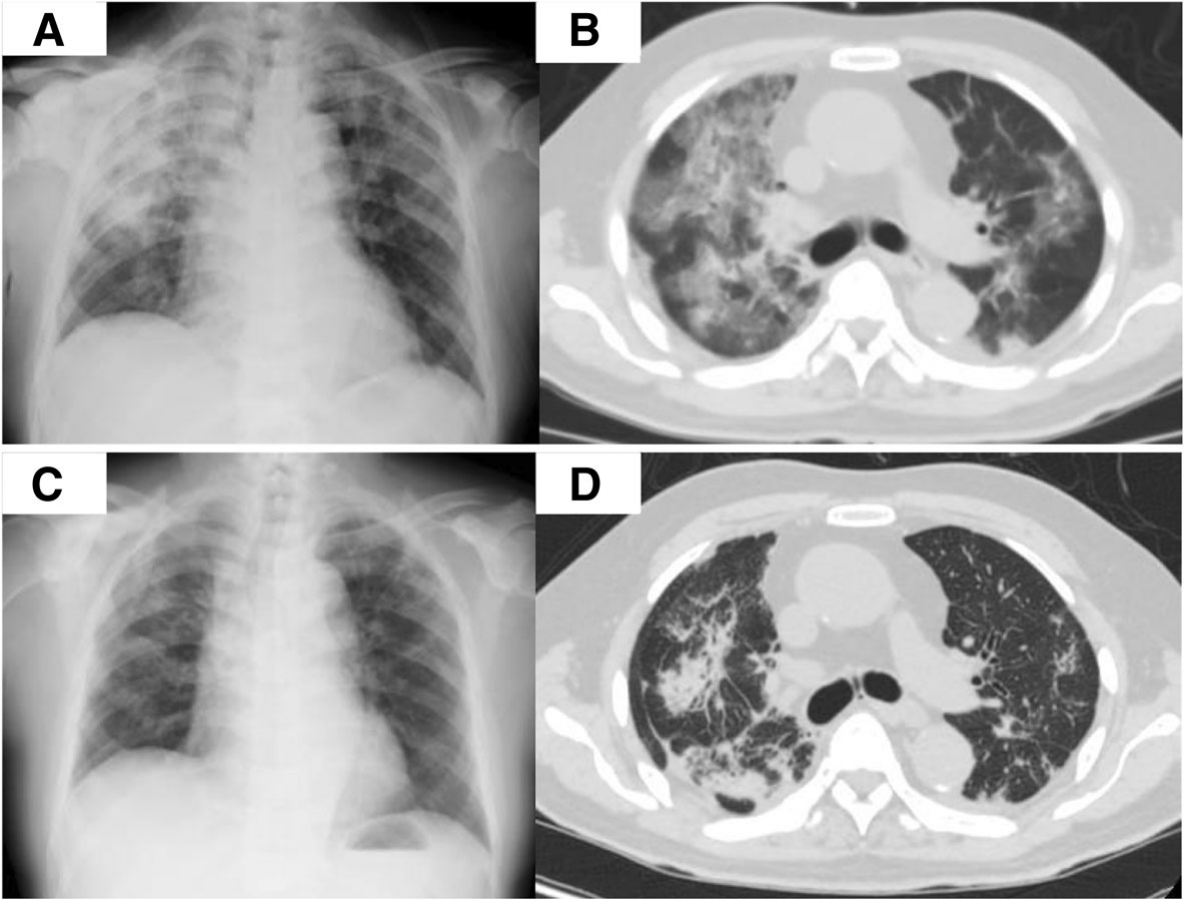
**a** Chest X-ray findings before the steroid pulse therapy. **b** Chest computed tomography findings before the steroid pulse therapy. **c** Chest X-ray findings after the steroid pulse therapy. **d** Chest computed tomography findings after the steroid pulse therapy

On admission in April 2017, the patient was started on cabazitaxel at 22.5 mg/m^2^ with proactive management of adverse events, including the use of a prophylactic long-acting granulocyte colony stimulating factor (G-CSF) and an oral empirical broad-spectrum antimicrobial agent (sitafloxacin hydrate, a new-generation, broad-spectrum oral fluoroquinolone that is very active against many Gram-positive, Gram-negative and anaerobic bacteria) to prevent febrile neutropenia. On day 10, grade 4 neutropenia and febrile neutropenia were observed. He was treated with an intravenous broad-spectrum antimicrobial agent (cefepime, a broad-spectrum cephalosporin with enhanced coverage against Gram-positive and Gram-negative bacteria), which resulted in cessation of the fever and increased neutrophil count, although sputum and blood cultures revealed negative. On day 18, he developed a secondary fever of 38 °C again despite neutrophil count of 3000/μL, so antimicrobial administration (cefepime) was restarted due to a suspected infection. Three days later, he complained of dyspnea and had a pulse oximetric saturation (SpO_2_) of 80% while breathing room air. The serum Krebs von den Lungen-6 (KL-6) and surfactant protein (SP-D) levels were 744 U/mL and 147 ng/mL (normal ranges, < 500 U/ml and < 100 ng/ml, respectively). The serum levels of procalcitonin, endotoxin concentration and (1,3)-beta-D-glucan were not elevated. Chest X-ray showed interstitial opacities indicative of inflammation in the irradiated field of the both lungs (Fig. 2a). Chest computed tomography (CT) showed interstitial lung disease with ground glass opacities in the irradiated field of both lungs (Fig. 2b). These characteristic image findings could have been associated with drug toxicity, due to the treatment of thoracic radiation therapy followed by cabazitaxel.

The intravenous steroid pulse therapy was started at a methylprednisolone of 1000 mg a day for 3 days, followed by prednisolone at 1.0 mg/kg of body mass per day for a week. The patient improved and dyspnea disappeared. SpO_2_ showed 95% while breathing room air. The ground-glass opacities had also improved (Fig. 2c, d). The dosage of prednisolone was gradually reduced every week. The follow-up observation is still ongoing.

## Discussion and conclusions

Cabazitaxel has yielded incremental extensions of survival for post-docetaxel patients. As Agarwal et al. mentioned, the success of cabazitaxel, following docetaxel, may warrant the continued evaluation of chemotherapeutic agents for metastatic CRPC (mCRPC) [2]. The common adverse events of cabazitaxel were diarrhea, fatigue, nausea, and neutropenia [3]. The most common clinically significant hematological grade 3 or higher adverse events of cabazitaxel were neutropenia (82%) and the incidence of dyspnea of grade 3 or higher was less than 1% with cabazitaxel in the TROPIC trial [4]. Cabazitaxel is a tubulin-binding taxane that blocks mitosis in tumor cells and could cause other minor side effects. We previously reported hemorrhagic cystitis in a patient who was treated with cabazitaxel [5]. An interstitial lung disease associated with docetaxel and paclitaxel was reported previously, however in this report, cabazitaxel could also cause a pulmonary interstitial disease after radiotherapy.

In our patient, the dose-reduction of cabazitaxel at a dose of 22.5 mg/m2 was required due to his reduced general condition. Prior treatment included docetaxel chemotherapy, enzalutamide, intravenous radium-223 and local radiotherapy. A full dose of cabazitaxel could induce severe adverse events in such a patient. A phase I cabazitaxel study in Japanese patients with mCRPC revealed the most frequent toxicities were neutropenia and febrile neutropenia, occurring at grade ≥ 3 in 100 and 54.5% of patients, respectively, although prophylactic administration of G-CSF was not permitted at cycle 1 [6]. Cabazitaxel induced a high rate of grade 3/4 neutropenia, which need to be proactively managed to avoid neutropenic complications. Di Lorenzo et al. revealed that prior cumulative dose was associated with reduced grade 3 to grade 4 neutropenia and anemia [7]. At our institute, all patients receive cabazitaxel at 20–25 mg/m^2^ administered intravenously on day 1 of each treatment cycle, together with prophylactic G-CSF. Of 41 patients with mCRPC treated with cabazitaxel in the previous retrospective study, grade ≥ 3 neutropenia and febrile neutropenia occurred in 21 and (53.6%) and 3 (6.8%) patients, respectively, which is believed to be an improvement compared to the phase I cabazitaxel study in Japanese patients [3]. Moreover, internal fixation of the left femur was performed to prevent pathological fractures due to metastasis to the left femur. Therefore, after consulting with the patient, we decided to use antibiotics prophylactically.

When the secondary high fever occurred, a multiple-drug-resistant bacterial infection was considered at first. However, CT image and laboratory tests did not indicate bacterial pneumonitis. They revealed drug induced interstitial lung disease, then a steroid therapy was started. Some antitumor drugs are associated with a recall effect of pneumonitis after radiotherapy, however, the etiology and mechanism is unknown yet [1]. The diagnosis of radiation recall pneumonitis induced by chemotherapy is established based on a history of chemotherapy after thoracic radiotherapy, clinical presentation, and radiographic abnormality. This report is a case of interstitial pneumonitis in a castration-resistant prostate cancer patient receiving cabazitaxel after thoracic radiation therapy and may help guide the management of patients with dyspnea and fever treated with cabazitaxel and radiotherapy.

## Data Availability

All data generated or analyzed during this study are included in this published article.

## Abbreviations

CRPC: Castration-resistant prostate cancer
CT: Computed tomography
G-CSF: Granulocyte colony stimulating factor
SpO_2_: A pulse oximetric saturation
